# Modernizing Regulatory Evidence with Trials and Real-World Studies

**DOI:** 10.1007/s43441-020-00131-5

**Published:** 2020-02-18

**Authors:** Nancy A. Dreyer, Marni Hall, Jennifer B. Christian

**Affiliations:** 1 IQVIA Real World Solutions, 201 Broadway, 5th Floor, Cambridge, MA 02139 USA; 2 IQVIA Real-World Solutions, Rockville, MD USA; 3 IQVIA Real World Solutions, Research Triangle Park, NC USA

We are taught that randomized controlled trials (RCTs) are the gold standard for evaluating whether a treatment can achieve its intended benefit because they are designed to isolate treatment effects while essentially balancing all other factors, known and unknown. While an elegant tool, the results from RCTs are not always generalizable to less idealized settings and more diverse patients. Real-world evidence (RWE), conducted alongside or as an extension of clinical trials, can play an important role in completing the picture of how well a therapy works, for which patients, and under what conditions. Such evidence has long been used for treatment and reimbursement decisions; the question at hand is whether, when and how RWE should contribute to regulatory decision-making and be considered credible enough to be counted as ‘substantial evidence of effectiveness.’ Among numerous efforts to evaluate RWE suitability for regulatory use, a recent Friends of Cancer Research (Friends) study provides valuable lessons for RCT and RWE zealots alike. Here, we review the complementary nature of RCTs and RWE, discuss the key learnings from the Friends project, and reinforce the call for continued examination of methods and data sources to guide reliable regulatory use of RWE for evaluating therapies.

## RCTs and RWE—Fundamental Differences and Complementary Potential

RCTs provide scientifically tidy comparisons, but they fall short in their utility for generalizability to more diverse patients, and complex conditions and treatments. By design, patients in RCTs are highly selected and cared for, with active encouragement to adhere to assigned treatments, close monitoring for disease progression, and optimal testing. The endpoints used in RCTs may not be directly relevant to patients and clinicians, especially surrogate endpoints used as substitutes for clinical outcomes that take a long time to develop [1]. For example, 36 of 54 new oncology treatments approved from 2008–2012 used surrogate endpoints for approval, but roughly four years after approval, only 14% showed improvement in overall survival (OS). Half (50%) of those newly approved drugs revealed no survival benefit, with no data available for the remainder [2].

In contrast, real-world data (RWD), an umbrella term for health data that are not collected in the context of highly controlled RCTs [3], captures information about benefits and risks for diverse patients and care settings under more typical conditions. RWE studies designed to supplement RCT results offer a scalable way to quantify benefits and risks that cannot be captured in the confines of most RCTs.

## Friends of Cancer Research Study Offers Key Lessons in the Value of RWE

Friends commissioned a group of stakeholders in 2018 to evaluate a series of real-world clinical endpoints for patients with advanced non-small cell lung cancer treated with immune checkpoint inhibitors, and to compare those results with RCTs. A common protocol intended to broadly match the RCT but with fewer inclusion and exclusion criteria was used by six “research-ready” RWD sources representing a range of care models including community oncology centers, health systems, academic medical centers, and integrated delivery system networks [4]. The tumor-based Response Evaluation Criteria in Solid Tumors (RECIST), a widely used measure in oncology RCTs for response to therapy, was not used due to a lack of sufficient detail in radiology reports and other data not regularly available in RWD [5]. Neither natural language processing nor artificial intelligence tools were used to mine unstructured data for this pilot study.

**Figure 1.**
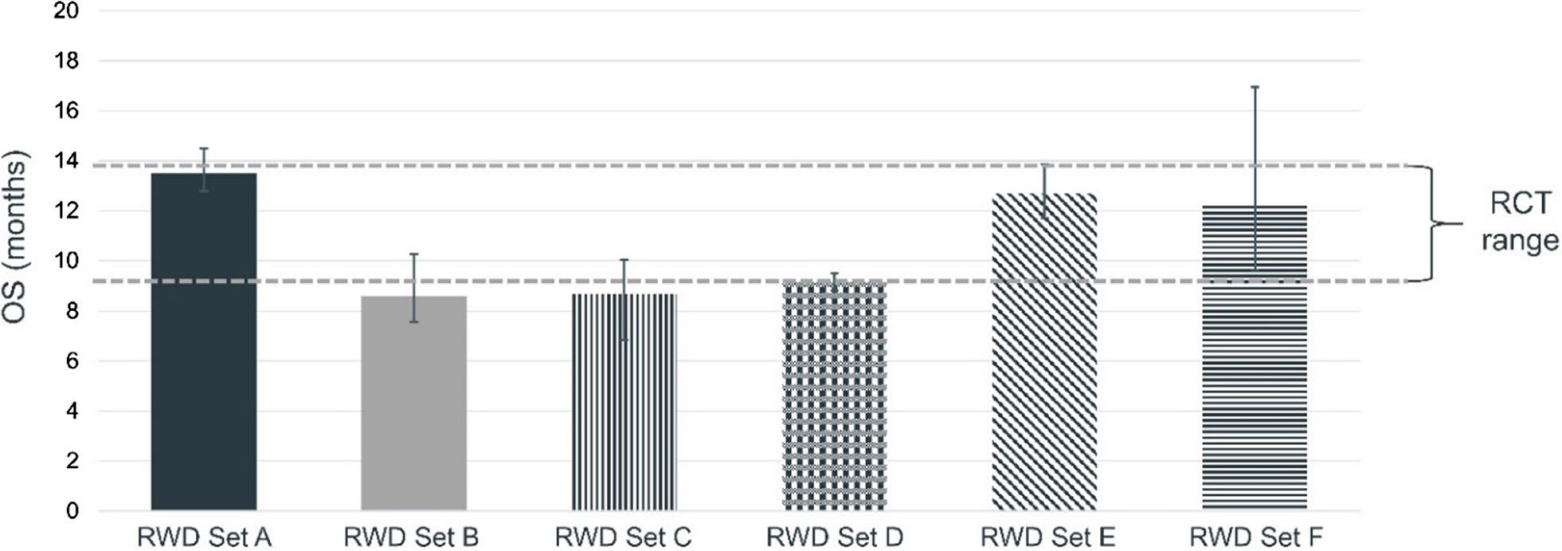
Comparing Overall Survival in RWD and RCT for Patients with Advanced Non-small Cell Lung Cancer Treated with Immune Checkpoint Inhibitors. Data sources include IQVIA, COTA, Flatiron, Kaiser Permanente Cancer Research Network, Mayo Clinic/Optum Labs, and The University of Iowa PCORnet.

**Figure 2.**
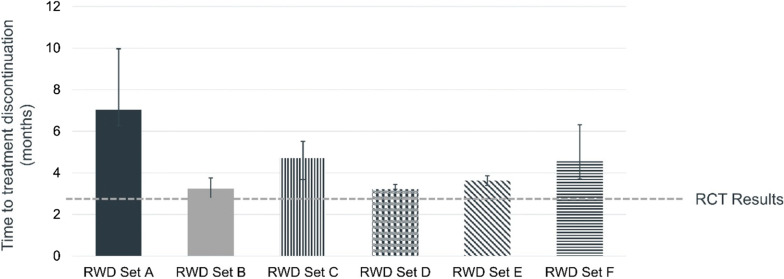
Comparing Time to Progression (Treatment Discontinuation) in RWD and RCT for Patients with Advanced Non-small Cell Lung Cancer Treated with Immune Checkpoint Inhibitors. Data sources include IQVIA, COTA, Flatiron, Kaiser Permanente Cancer Research Network, Mayo Clinic/Optum Labs, and The University of Iowa PCORnet.

The endpoints studied were generally consistent across RWD sources and were close to what was seen in RCTs that examined the same endpoints in patients with NSCLC. For OS, where RWD estimates were outside the 95% confidence limits observed in a meta-analysis of RCTs, they were short by only two weeks (a lower limit of 8.6 months vs. 9 months), which may reflect the true range of survival seen in RW settings (Fig. 1). Interestingly, time to progression measured by changes in treatment were always slightly longer in RW setting (Fig. 2). This finding likely reflects the common RW practice of treating beyond progression, as well as differential methods for detecting progression and the variability that occurs in the absence of following a single common trial protocol for when treatment should be discontinued. Taken as a body of evidence, the RCTs demonstrated that immune checkpoint inhibitors are efficacious in optimal settings, and RWE provided realistic estimates of effectiveness in routine care settings. The key lessons here are that (1) RWD can generate meaningful evidence that is relevant to understanding how medical products perform in non-research, routine care, and (2) results from RWD can be different from RCT but not necessarily wrong. While there may be variations in the way data are collected, recorded, and ultimately in the findings from RWD, they may be sufficient and informative to support both clinical and regulatory decision-making.

## Additional Efforts to Expand Routine Use of RWD and RWE

Several other formative activities are underway to advance the use of RWD and RWE [6, 7], though there remains much debate as to what validation exercises should be required for each new data endpoint, dataset and data source. For example, the FDA’s Digital Health Software Pre-Certification program, currently in a pilot phase, is aimed at creating a regulatory environment that is both efficient and effective in utilizing software as a medical device [8], a strong indication that systematic use of RWD generated from sensors, wearables, and other personalized devices is inevitable. The FDA MyStudies software application, designed to facilitate the use of patient-reported data that can be linked to EMR and other data, further signals a world where patient-generated health data will be used for regulatory decision-making. [9] The European Commission through its Innovative Medicine Initiative conducted a study of patient-generated data which showed that patients were largely accurate reporters of prescription medications and also provided valuable information about the use of non-prescription medications, recreational drugs, alcohol and tobacco, giving credence to the utility of well-designed direct-to-patient approaches [10]. More recently, the European Medicines Agency and the Heads of Medicines Agency have released two reports showing their deep interest in Big Data (aka real-world data) including ideas for necessary changes in infrastructure to facilitate best use [11, 12].

The Duke Margolis Center for Health Policy Methods Working Group contributed to this body of evidence by mapping the frequently used RWE methods to the characteristics of an adequate and well-controlled study as outlined in the US Code of Federal Regulations [13]. Once credibility of the findings are established, they argue, as we do, that RWE can be complementary to other sources of information and should contribute to the totality of accruing evidence to support a regulatory decision. [14]

## Discussion

We simply cannot learn everything we need to know to advance the use of new medical products through RCTs alone. In fact, we know little about risks and benefits when these products are first on the market due, in part, to the increased complexities of treatment regimens and the lack of transparency and generalizability of clinical trial results [15, 16]. Delivery of care is complex and a diversity of approaches to generating evidence should be embraced to maximize what can be learned and how it can inform healthcare and regulatory decisions [17]. Given the complexities of health systems, delivery of health care, and the high degree of variation in human responses to treatment, we should expect that results from RWE may differ from RCTs yet still be correct, as the Friends results illustrate.

Understanding in which circumstances RWD can be used for reliable inference requires careful consideration of many factors on a study-by-study basis [18]. While qualification of study designs, analytic methods and data sources has merit, we should not wait for an entirely codified approach before expanding application of RWD to guide decisions about effectiveness, especially since the majority of drug approvals are now accelerated in one way or another. As confidence grows through various demonstration projects like those discussed here, we can sensibly integrate these innovations into our well-established arsenal of clinical evidence generation approaches and use the totality of evidence to inform health care and regulatory decisions. As consensus develops around data quality metrics and documentation for data provenance becomes routine, the health care ecosystem will be better equipped to triage and make good use of the tsunami of health-informing data generated each day.
